# Simpson's Paradox, Lord's Paradox, and Suppression Effects are the same phenomenon – the reversal paradox

**DOI:** 10.1186/1742-7622-5-2

**Published:** 2008-01-22

**Authors:** Yu-Kang Tu, David Gunnell, Mark S Gilthorpe

**Affiliations:** 1 Biostatistics Unit, Centre for Epidemiology & Biostatistics, University of Leeds, 30/32 Hyde Terrace, Leeds, LS2 9JT, UK; 2 Leeds Dental Institute, University of Leeds, Clarendon Road, Leeds, LS2 9LU, UK; 3 Department of Social Medicine, University of Bristol, Bristol, BS8 2PR, UK

## Abstract

This article discusses three statistical paradoxes that pervade epidemiological research: *Simpson's paradox*, *Lord's paradox*, and *suppression*. These paradoxes have important implications for the interpretation of evidence from observational studies. This article uses hypothetical scenarios to illustrate how the three paradoxes are different manifestations of one phenomenon – the *reversal paradox *– depending on whether the outcome and explanatory variables are categorical, continuous or a combination of both; this renders the issues and remedies for any one to be similar for all three. Although the three statistical paradoxes occur in different types of variables, they share the same characteristic: the association between two variables can be reversed, diminished, or enhanced when another variable is statistically controlled for. Understanding the concepts and theory behind these paradoxes provides insights into some controversial or contradictory research findings. These paradoxes show that prior knowledge and underlying causal theory play an important role in the statistical modelling of epidemiological data, where incorrect use of statistical models might produce consistent, replicable, yet erroneous results.

## Introduction

This article discusses three statistical paradoxes that pervade epidemiological research: *Simpson's paradox*, *Lord's paradox*, and *suppression*. These paradoxes are not just tantalising puzzles of purely academic interest; potentially, they have serious implications for the interpretation of evidence from observational studies. Scenarios which are associated with and can be explained by these paradoxes are discussed. A concise explanation of these paradoxes and an historical overview is also provided. Simulated data based upon the foetal origins of adult diseases hypothesis [1,2] are used to illustrate how the three paradoxes are different manifestations of one phenomenon – *the reversal paradox *– depending on whether the outcome and explanatory variables are categorical, continuous or a combination of both; this renders the issues and remedies for any one to be similar for all three. All statistical analyses were performed within SPSS 15.0 (SPSS Inc, Chicago, USA).

## Foetal origins hypothesis

The 'foetal origins of adult disease' hypothesis (FOAD), which has evolved into the 'developmental origins of health and disease' (DOHaD) hypothesis [1,2], was proposed to explain the associations observed between low birth weight and a range of diseases in later life. These associations have been interpreted as evidence that growth retardation *in utero *has adverse long-term effects on the development of vital organ systems which predispose the individuals to a range of metabolic and related disorders in later life. Nevertheless, although an inverse association between birth weight and disease in later life was found in some studies, this relationship was only established in many studies after the current body size variables such as body mass index (BMI), body weight and/or body height were adjusted for in the regression analysis. As body sizes may be in the causal pathway from birth weight to health outcomes in later life, the justification of this adjustment of current body sizes has been questioned recently [3-8].

**Figure 1:**
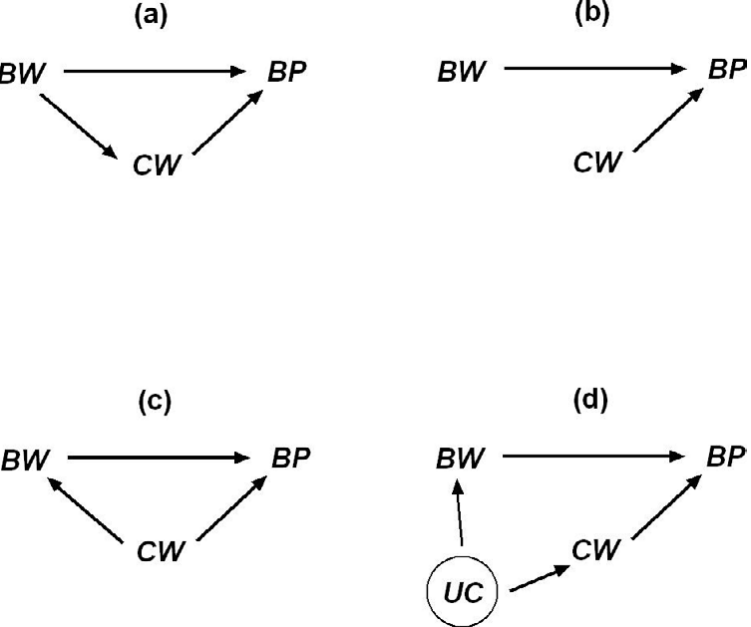
Causal models expressed as directed acyclic graphs for possible relationships between the three observed variables: birth weight (*BW*), current body weight (*CW*) and systolic blood pressure (*BP*). *UC *is an unobserved variable that affects both *BW *and *CW*. In Figure 1d, there is a back-door path from *BP *to *BW *via *CW *and *UC*, so the association between *BP *and *BW *is therefore biased. The adjustment of *CW *can block the backdoor path from *BP *to *BW *via *UC*.

Using the inverse relationship between birth weight and systolic blood pressure in later life as an example, Figure 1 shows the directed acyclic graphs [9-11] for the possible relationships between the three observed variables: birth weight, current body weight and systolic blood pressure. In Figure 1a, current body weight is on the causal pathway from birth weight to systolic blood pressure, so current body weight is not a genuine confounder and should not be adjusted for. In Figure 1b, there is no relationship between birth weight and current body weight, and therefore the latter is not a confounder for the relationship between birth weight and blood pressure either. However, this model cannot explain the observed positive correlations between birth weight and current body weight in many epidemiological studies. In Figure 1c, current body weight is a confounder because it is ancestor to both birth weight and blood pressure in the directed acyclic graph [9-11]. Obviously, this scenario is implausible in reality because current body weight cannot affect birth weight. In Figure 1d, the observed positive correlation between birth weight and current body weight is due to an unobserved confounder, UC, which affects both birth weight and current body weight. Also, there is no path from birth weight and current body weight [7], i.e. if UC could be identified and measured, birth weight and current body weight would be independent, conditional on UC [12]. More complex causal diagrams for the three variables are possible by incorporating more unobserved variables in the model. However, the four scenarios in Figure 1 are sufficient for our discussion in this study, so we do not pursue them further.

Figure 1a,c and 1d all explain the observed correlation structure amongst birth weight, current body weight and blood pressure equally well, and it is not possible to judge which one is true based upon the observed data. For example, researchers may argue current body weight is a genuine confounder in Figure 1d and therefore should be adjusted for [7]. This can only be confirmed when the unobserved confounder (be parental, genetic, or environmental factors) is identified *and *the conditional independence between birth weight and current weight is satisfied.

Nevertheless, the adjustment of current body weight in the statistical analysis will change the estimated relationship between birth weight and blood pressure, as the adjusted relationship is a conditional relationship. Differences between the unadjusted and adjusted (i.e. unconditional and conditional) relationships frequently cause confusion in the interpretations of statistical analyses and they also give rise to three statistical paradoxes, which we shall explain in the next section.

## Simpson's Paradox

Simpson's paradox [13], or Yule's paradox [14], is a well known statistical phenomenon. It is observed when the relationship between two categorical variables is reversed after a third variable is introduced to the analysis of their association, or alternatively where the relationship between two variables differs within subgroups compared to that observed for the aggregated data. Although first discussed by Karl Pearson in 1899 [15], it is George Udny Yule, once Pearson's assistant, who provides a detailed assessment of this problem in 1903 [14].

### A numerical example

Table 1 provides a summary of a hypothetical survey of 1000 adult males in England based on data simulated using values derived from the literature [16] and surveys conducted by the UK Department of Health [17]. Data are simulated such that the three variables systolic blood pressure (*BP*), birth weight (*BW*), and current body weight (*CW*) are positively correlated: the correlation between *BP *and birth weight (*r*_*BW*-*BP*_) is weak (0.11); whereas the correlations between birth weight and current weight (*r*_*BW*-*CW*_) and between current weight and *BP *(*r*_*CW*-*BP*_) are reasonably strong (0.52 and 0.50, respectively).

**Table 1:** Summary of the analysis of simulated systolic blood pressure, birth weight and current body weight data for 1000 adult males

	**N**	**Minimum**	**Maximum**	**Mean**	**Standard Deviation**
**Current weight (kg)**	1000	38.02	127.08	82.69	14.61
**Systolic BP (mmHg)**	1000	89.36	168.88	129.78	11.14
**Birth weight (kg)**	1000	1.37	5.42	3.51	0.63

Suppose the research question is to investigate whether or not there is an association between low birth weight and high blood pressure in later life. In this hypothetical study, low birth weight is defined as birth weight lower than the population mean (i.e. < 3.5 Kg), and high blood pressure is defined as systolic *BP *greater than the mean value (i.e. > 135 mmHg). The results are summarized in Table 2. It is noted that the probability of developing high blood pressure is 0.272 for subjects with low birth weight and 0.362 for subjects with high birth weight. This indicates that low birth weight has a protective effect of developing high blood pressure. However, when these subjects are stratified according to their current weight (> 90 Kg vs. < = 90 Kg), the risk of developing high blood pressure is consistently higher amongst subjects with low birth weight compared to those with high birth weight. It seems to be quite counter-intuitive that low birth weight has an adverse effect on blood pressure for both subgroups of current weight, yet a protective effect on the groups as a whole.

**Table 2:** Numbers and Percentages of subjects with high blood pressure (> 135 mmHg) according to their birth weight and current body weight

	**Normal BP**	**High BP**	**Total**	**Percentage of subjects with high *BP***
***Overall:***				
**Low birth weight**	354	132	486	27.2%
**High birth weight**	328	186	514	36.2%
**Total**	682	318	1,000	31.8%

**Current weight < = 90 Kg**				
**Low birth weight**	329	99	428	23.1%
**High birth weight**	221	55	276	19.9%
**Total**	550	154	704	21.9%

**Current weight > 90 Kg**				
**Low birth weight**	25	33	58	56.9%
**High birth weight**	107	131	238	55.0%
**Total**	132	164	296	55.4%

### Interpretation

**Figure 2:**
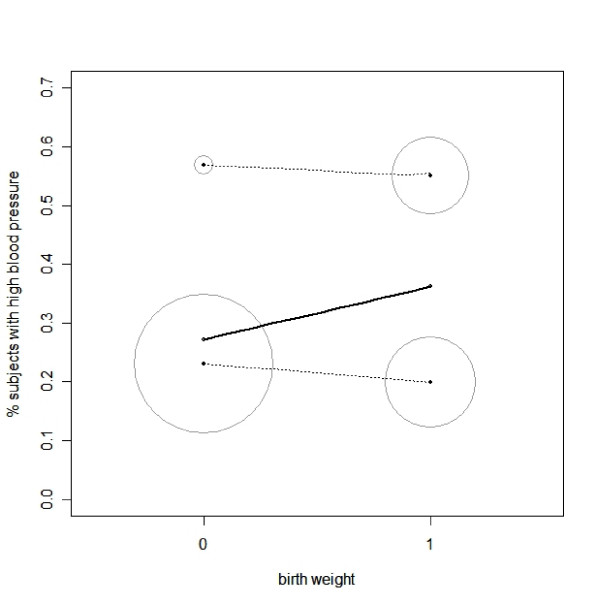
Graphical representation of Simpson's paradox. The two circles on the top of the panel represent subjects with lower (0) and higher birth weight (1), respectively, in the subgroup with current weight > 90 Kg. The two circles on the bottom of the panel represent subjects with lower (0) and higher birth weight (1) in the subgroup with current weight ≤ 90 Kg. The area of the circles is proportional to the sample size of the subgroups they represent. The two dotted lines show that subjects with lower birth weight have a higher risk of developing high blood pressure in each current weight subgroups. However, as a whole group, subjects with lower birth weight have a lower risk of developing high blood pressure (the black solid line).

In this scenario, there are substantial differences in the numbers of subjects with low birth weight between the two subgroups of current weight, because lower birth weight babies on average are smaller in adulthood. Therefore, the overall relation between low birth weight and high blood pressure is a sum of weighted relations between the two variables in each subgroup. A graphical representation of this paradox, first proposed by Paik [18], is given in Figure 2. Due to a greater influence of the lower risk of developing high blood pressure in the subjects with low birth weight and lower current weight, the adverse relation is reversed in the whole-group analysis (solid line in Figure 2). Note that, in the following two scenarios, the adjustment for current weight will not change the relationship between birth weight and *BP *[12], if: (a) there is no difference in the percentages of subjects with high current weight between the two subgroups of birth weight (i.e. no correlation between birth weight and current weight); or (b) there is no association between *CWb *and *BP *in the subgroups stratified by *BWb *(i.e. the association between *BP *and current weight is entirely caused by the association between birth weight and *BP*). The problem is whether the relation between low birth weigh and high blood pressure in the whole group provides an answer to the intended research question, or whether the relation in the two subgroups does this. In other words, should *CWb *be considered a confounder and hence adjusted for in the statistical models?

In statistical language, adjustment for current body weight represents a *conditional *relationship; the relationship between birth weight and blood pressure is *conditional *on current body weight. Although there are substantial differences in the numbers of subjects with low birth weight between the two subgroups of current weight, the adjustment for *CWb *indicates that *if *all subjects had the same level of current body weight, subjects with low birth weight would have a greater risk of developing high blood pressure, i.e. the adjustment of *CWb *erases the greater influence of subjects with low birth weight and lower current weight on the association between birth weight and blood pressure, as people born smaller in general grow into a smaller adults.

Simpson's paradox has broad implications for epidemiological research since it indicates that making causal inference from any non-randomised study (e.g. cohort studies, case-control studies) can be difficult, because, whilst it is possible to control for the differences between cases and controls, there will always be the possibility that an unobserved and therefore unadjusted confounder might attenuate the association (or even reverse its direction) between exposure and outcome, due to the difference in the mean values or the distribution of confounders between the case or control group. Nevertheless, whether or not there is any unobserved (and therefore unadjusted) confounder may not always be an issue of debate, because in most epidemiologic studies, the important confounders are generally known. The controversy in making causal inference arises in situations where the adjusted variable may not be a genuine confounder [6,7,19,20]. Within epidemiology, Simpson's paradox is closely linked to the concepts of confounding [9] and incollapsibility [10].

## Lord's Paradox

Lord's paradox was named after two short articles in the psychology literature by Frederick M Lord regarding the use of analysis of covariance (ANCOVA) within non-experimental studies [21,22]. In contrast to Simpson's paradox, little discussion of Lord's paradox can be found in the statistical and epidemiological literature [23], though social scientists have shown a great interest in this phenomenon [24-28]. Lord's paradox refers to the relationship between a continuous outcome and a categorical exposure being reversed when an additional continuous covariate is introduced to the analysis. One specific example is that the additional covariate is a measure made at baseline within a longitudinal study, where the outcome is the same variable measured some time later (e.g. following an intervention). Therefore, the aim is to measure change in the outcome by adjusting for the baseline measurements, and the categorical covariate might be the exposure/control groups – this is the familiar design for ANCOVA. This controversy was first discussed in 1910 between Karl Pearson and Arthur C Pigou when they debated the role of parental alcoholism and its impact on the performance of children [29].

### A numerical example

Considering the previous numerical example for Simpson's paradox, we examine current body weight (*CW*) and blood pressure (*BP*) as continuous variables, retaining birth weight as a binary (*BWb*). The two-sample *t*-test shows that, on average, the blood pressure of subjects with higher birth weight is 2.49 mmHg (95%CI: 1.12, 3.87) *greater *than those with lower birth weight. However, using ANCOVA (i.e. linear regression with a (categorical) group-allocating variable and with the adjustment of a continuous confounding variable), adjusting for current weight as a covariate, the blood pressure of subjects with higher birth weight becomes 2.94 mmHg (95%CI: 1.12, 3.87) *lower *than those with lower birth weight.

### Interpretation

Differences in the results of the two analyses are due to adjustment in the second analysis for current body weight (*CW*). As current weight is positively associated with both *BP *and *BWb*, it is expected that the relation between *BP *and *BWb *will change when current weight is adjusted for. In randomised controlled trials, mean values of the adjusted baseline covariate are expected to be approximately equal across treatment and control groups since, assuming randomisation has been achieved, baseline variation should be *within *groups rather than *between *groups), i.e. there is no correlation between the group variable and adjusted covariate (i.e. in our numerical example, no correlation between *BWb *and current weight). In such circumstances it is well known that using ANCOVA achieves the same estimated treatment difference across groups as found by the *t*-test, though the former will generally have greater power [30,31]. Recall our previous discussion of two scenarios in the section on Simpson's paradox, where the adjustment for *CWb *will not change the relationship between *BWb *and *BP*. Randomised controlled trials may thus be seen as a special case of scenario (a) where there is no difference in the mean current weight between the two sub-groups of birth weight.

**Figure 3:**
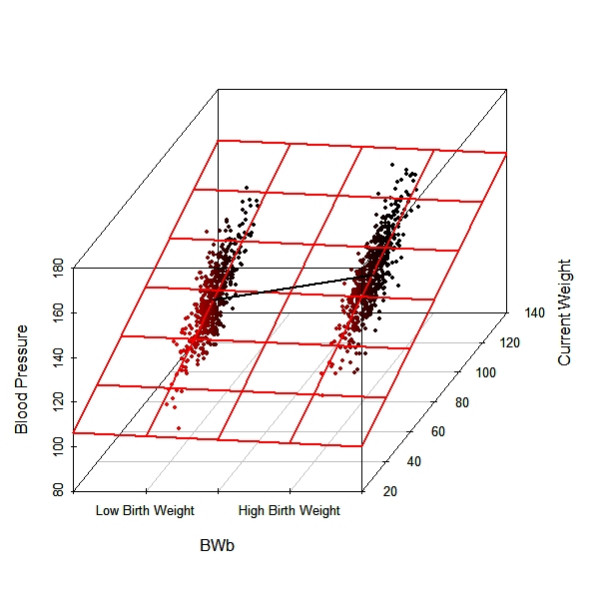
A 3-dimensional scatter plot for the numerical example in Lord's paradox. The solid line shows that the mean blood pressure of subjects with higher birth weight (*BWb *= 1) is greater than those with lower birth weight (*BWb *= 0). However, at each level of current weight, the mean blood pressure of subjects with higher birth weight is lower than those with lower birth weight (the horizontal red lines).

Figure 3 is a three-dimensional representation of the associations amongst the three variables. Although the solid black line shows that subjects with higher birth weight (coded as 1) have on average a greater blood pressure than those with lower birth weight (coded as 0), the various horizontal red lines with a negative slope indicate that at each level of current weight, subjects with higher birth weight have a lower mean blood pressure than those with lower birth weight.

In statistical language, results from the regression analyses are *conditional *on both birth weight groups having equal mean current weight in later life, and if true there would be a benefit from low birth weight in terms of blood pressure. However, since the two groups have a different mean current weight in later life, results from the regression analysis need to be interpreted with caution. In Simpson's paradox, the discussion surrounds the differences in results between unconditional and conditional risk/probability, and in Lord's paradox, discussion is around the differences in results between unconditional and conditional means.

## Suppression

Of the three paradoxes, suppression effects within multiple regression are probably the least recognised amongst clinical and epidemiological researchers, though the suppression phenomenon has been extensively discussed by statisticians [32-34] and methodologists from the social sciences [35,36]. The classical definition of suppression is that a potential covariate that is unrelated to the outcome variable (i.e. has a bivariate correlation of zero) increases the overall model fit within regression (as assessed by *R*^2^, for instance) when this covariate is added to the model. This seems counter-intuitive and needs some explanation.

Suppose *y *is the outcome variable, and *x*_1_and *x*_2 _are two covariates (i.e. 'explanatory' variables). Denote the bivariate Pearson correlation between *y *and *x*_1 _as *r*_*y*1_; the correlation between *y *and *x*_2 _as *r*_*y*2_; and the correlation between *x*_1 _and *x*_2 _as *r*_12_. Within multiple regression, where *y *= *b*_0 _+ *b*_1 _*x*_1 _+ *b*_2 _*x*_2_, the standardized partial regression coefficients of *b*_1 _and *b*_2 _for *x*_1 _(*β *_1_) and *x*_2 _(*β *_2_), respectively, are given by [37]:

$$\begin{array}{ccc} \beta_{1}=\frac{r_{y1}-r_{12}r_{y2}}{1-r_{12}^{2}} & \& & \beta_{2}=\frac{r_{y2}-r_{12}r_{y1}}{1-r_{12}^{2}} \end{array}.$$

Now suppose that *y *is adult blood pressure (*BP*), *x*_1 _birth weight (*BW*), and *x*_2 _adult current weight (*CW*). Many studies have shown the bivariate correlation (*r*_*y*1_) between *BP *(*y*) and birth weight (*x*_1_) to be negative though weak [38,39], whilst others show this to be positive [40]; for illustrative purposes only, assume that *r*_*y*1 _is zero. Many studies show the bivariate correlation (*r*_*y*2_) between *BP *(*y*) and current weight (*x*_2_) to be positive [41]. When *BP *is regressed on birth weight and current weight, the model fit assessed by *R*^2 ^becomes [37]:

*R*^2 ^= *r*_*y*1 _* *β *_1 _+ *r*_*y*2 _* *β *_2_.

Since *r*_*y*1 _is equal to zero, equation (2) becomes:

$$R^{2}=r_{y2}*\beta_{2}=\frac{r_{y2}^{2}}{1-r_{12}^{2}}.$$

Since $1-r_{12}^{2}$ will always be smaller than 1, $R^{2}=\frac{r_{y2}^{2}}{1-r_{12}^{2}}$ will always be greater than $r_{y2}^{2}$. By including *x*_1 _in the regression model, more variance of *y *is 'explained', i.e. the predictability of the model increases. However, this seems counterintuitive, since the zero bivariate correlation between *y *and *x*_1 _(*r*_*y*1 _= 0) indicates that no more variance in *y *can be explained by *x*_1_. So where does the additional 'explained variance' in *y *come from when *x*_1 _is entered in the regression model? The answer is that the additional explained variance in *y *comes from *x*_2_.

Although *x*_1 _is not correlated with *y*, it is positively correlated with *x*_2_, which in turn is positively correlated with *y*. When *x*_1 _is entered in the model, it 'suppresses' the part of *x*_2 _that is uncorrelated with *y*, thereby increasing overall predictability. In other words, the role of *x*_1 _in the model is to suppress (reduce) the noise (the uncorrelated component of *x*_2_) within the correlation between *y *and *x*_2_, as though any uncertainty in *x*_2 _'predicting' *y *is 'explained' by *x*_1_.

It is not only *R*^2 ^that is increased; the coefficient for *x*_2_, $\beta_{2}=\frac{r_{y2}}{1-r_{12}^{2}}$, becomes greater than *r*_*y*2_. Furthermore, although *r*_*y*1 _is equal to zero, *β*_1 _is not zero and becomes negative: $\beta_{1}=\frac{-r_{12}r_{y2}}{1-r_{12}^{2}}$. In general, the greater the positive correlation between *x*_1 _and *x*_2_, the greater the absolute value of *β*_1 _and *β*_2_. However, having *r*_*y*1 _equal zero (or being negative) is not necessary to observe suppression; *r*_*y*1 _may be positive and *x*_1 _may still be a suppressor [35].

It was Paul Horst, in 1941, who first explored this curious phenomenon within educational research [42], and in the last few decades, many statisticians have been interested in this topic [33-35]. There are still very few discussions within the clinical and epidemiological literature regarding the impact of suppression (i.e. the impact on the changes in the regression coefficients and *R*^2^) on the interpretation of non-randomised studies whilst making statistical adjustment for covariates within regression [12,43].

### A numerical example

Considering the previous numerical examples for Simpson's paradox and Lord's paradox, all three variables are now treated as continuous. Simple regression shows a positive association between *BP *and birth weight: the regression coefficient for birth weight is 1.861 mmHg/Kg (95% CI: 0.770, 2.953). Simple regression also reveals a positive association between *BP *and current weight: the regression coefficient for current weight is 0.382 (95% CI = 0.341, 0.423) mmHg/Kg. Following the practice of many previous studies, *BP *is regressed on birth weight and current weight simultaneously and the partial regression coefficients for birth weight and current weight are -3.708 (95% CI = -4.794, -2.622) and 0.465 (95% CI = 0.418, 0.512) mmHg/Kg respectively, and both are highly statistically significant (Table 3). Thus, after adjusting for current weight, birth weight has a significant inverse association with *BP*, suggesting that hypertension is associated with lower birth weight.

**Table 3:** Simple and multiple regression models for simulated hypothetical data on birth weight (*BW*), blood pressure (*BP*), and current body weight (*CW*); the dependent variable in all three models is *BP*.

**Model**	**Regression Coefficients (Standard Errors)**		**Standardised Coefficients**	***P*-values**	***R^2^***
**1**	**Intercept**	123.258 (1.981)		(< 0.001) ^†^	0.011
	**Birth weight**	1.861 (0.556)	-0.105	0.001	
**2**	**Intercept**	98.173 (1.755)		(< 0.001)^†^	0.251
	**Current weight**	0.382 (0.021)	0.501	< 0.001	
**3**	**Intercept**	104.330 (1.948)		(< 0.001)^†^	0.283
	**Birth weight**	-3.708 (0.553)	-0.210	< 0.001	
	**Current Weight**	0.465 (0.024)	0.610	< 0.001	

^† ^It is irrelevant to formally test the intercept for statistical significance in this instance.

It is noteworthy that not only the association of birth weight with *BP *is reversed (coefficients change from 1.861 to -3.708 mmHg/Kg), but that the impact of current weight also increases from 0.382 to 0.465 mmHg/Kg. The *R*^2 ^for multiple regression is 0.283, which is greater than the sum of the squared correlations for birth weight ((0.105)^2 ^= 0.011) and current weight ((0.501)^2 ^= 0.251), i.e. 0.262. Therefore, the explained variance of *BP *is greater than the sum of the explained variances for the two simple regression models.

**Figure 4:**
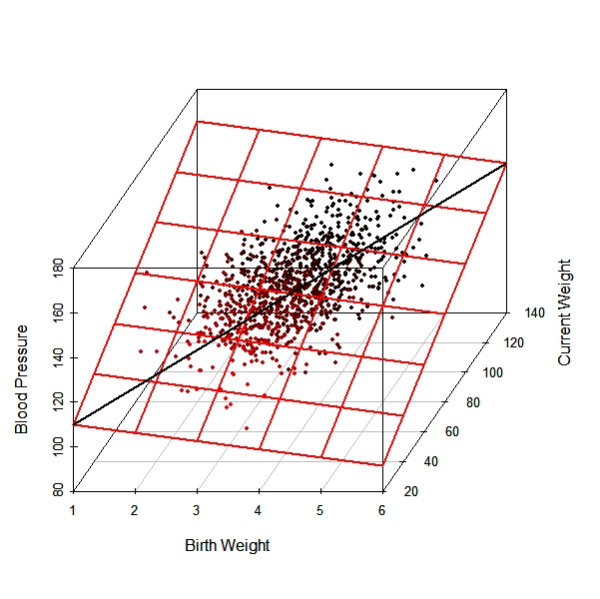
A 3-dimensional scatter plot for the numerical example of suppression. The solid black line shows that the marginal relation between *BP *and birth weight is positive. However, the conditional relation between *BP *and birth weight (conditional on current weight) is reversed (the horizontal red lines).

Figure 4 is a three-dimensional representation of the associations amongst the three continuous variables. Although the solid black line shows that birth weight has a positive association with blood pressure, the various horizontal red lines with a negative slope indicate that at each level of current weight, birth weight has an inverse relationship with blood pressure.

### Interpretation

In the hypothetical foetal origins example, the strength of association between *BP *and birth weight differs considerably between simple regression and multiple regression. Which model genuinely reflects their true causal relationship depends on whether or not current weight should be adjusted for; whether or not current weight is a confounder for the relationship between *BP *and birth weight, which depends upon biological and clinical knowledge, not *ad hoc *statistical analyses and changes in the estimated effects [11]. The question is whether or not it is also biologically and clinically feasible to isolate the independent effect of birth weight on *BP *by removing the impact of current weight on *BP *[3,5-7,44]. In other words, changes in the regression coefficient for birth weight caused by current weight being adjusted for in multiple regression is irrelevant to whether or not current weight is viewed to be a confounder. The definition of confounding depends upon the *a priori *causal model assumed by the investigator [8,11], which then dictates which statistical model is adopted.

In statistical language, results from adjustment for current weight are *conditional *on all babies growing to the same size in adulthood. In Simpson's paradox, the 'paradox' is due to differences in the results between unconditional and conditional risk/probability, and in Lord's paradox, it is due to differences in the results between unconditional and conditional means. In suppression, the paradox is due to differences in the results between the marginal (i.e. unconditional) *BP-*birth weight relation and the *BP-*birth weight relation conditional on current weight.

## Discussion

The reversal paradox is often used as the generic name for *Simpson's paradox*, *Lord's Paradox*, and *suppression *(see Table 4). Whilst the original definition and naming of the reversal paradox was derived from the notion that the direction of a relationship between two variables might be reversed after a third variable is introduced, this nevertheless may generalise to scenarios where the relationship between two variables is enhanced, not reduced or reversed, after the third variable is introduced (as with many studies on the foetal origins hypothesis).

**Table 4:** Comparison of Simpson's paradox, Lord's Paradox, and suppression

**Type of Reversal Paradox**	**Outcome (*illustrated example*)**	**Exposure (*illustrated example*)**	**Covariate/'Confounder' (*illustrated example*)**
**Simpson's Paradox**	Categorical (*hypertension*)	Categorical (*birth weight: high vs. low*)	Categorical (*current weight: high vs. low*)
**Lord's paradox**	Continuous (*blood pressure*)	Categorical (*birth weight: high vs. low*)	Continuous (*current weight*)
**Suppression**	Continuous (*blood pressure*)	Continuous (*birth weight*)	Continuous (*current weight*)

In non-randomised studies, the reversal paradox can often occur due to 'controlling' for what is typically termed a confounder, even though a clear definition of what is meant by 'confounder' is rarely provided (contingent on understanding its role in the biological/clinical process being modelled). Differences in the strength or even direction of any association between outcome and exposure might give rise to contradictory interpretations regarding potential *causal *relationships. Furthermore, it is very difficult, if not impossible, to compare results across studies where many varied attempts are made to control for different confounders, especially in the absence of any consistent reasoning given for the choice of confounders. In some situations, statistical adjustment might introduce bias rather than eliminate it [45].

It might be suggested that the adjustment of current weight in our foetal origins example can be viewed as estimations of direct and indirect effects, such as those in path analysis or structural equation modelling. Recall Figure 1a, the path from birth weight to BP is to estimate the direct effect of birth weight → BP, and then the path from birth weight → current weight → BP is to estimate the indirect effect. For instance, in the model 3 of Table 3, the regression coefficient for birth weight, -3.708, is the direct effect, and the indirect effect is derived from 0.465 (the regression coefficient for current weight in model 3) multiplied by 11.976 (the simple regression coefficient for birth weight when current weight is regressed on birth weight) = 5.569. The total effect is therefore -3.708 + 5.569 = 1.861, which is the simple regression coefficient for birth weight in the model 1 of Table 3. Our reservation with interpreting the results from model 3 as the partition of the total effect into direct and indirect effect is that many variables, such as current height and current BMI, can be put in between birth weight and BP, and it can be claimed that there is more than one indirect effect. Furthermore, any body size measured after birth, for example, body weight at year one, year two etc, can be adjusted for in the model and presumably used to estimate the indirect effects and direct effect. Whilst the total effect of birth weight on BP is not affected by the numbers of intermediate body size variables in the model, the estimation of 'direct' effect differs when different intermediate variables are adjusted for. Unless there is experimental evidence to support the notion that there are indeed different paths of direct and indirect effects from birth weight to BP, we are cautious of using such terminology to label the results from multiple regression, as with model 3. In other words, to determine whether the unconditional or conditional relationship reflects the true physiological relationship between birth weight and blood pressure, experiments in which birth weight and current weight can be manipulated are required in order to estimate the impact of birth weight on blood pressure.

Although the three statistical paradoxes occur in different types of variables, they share the same characteristic: the association between two variables can be reversed, diminished, or enhanced when another variable is statistically controlled for. Understanding the concepts and theory behind these paradoxes will provide insights into some of the controversial or contradictory results from previous research. Prior knowledge and theory play an important role in the statistical modelling of non-randomised data. Incorrect use of statistical models might produce consistent, replicable, yet erroneous results.

## Competing interests

The author(s) declare that they have no competing interests.
